# Direct Purification of Digestate Using Ultrafiltration Membranes: Influence of Pore Size on Filtration Behavior and Fouling Characteristics

**DOI:** 10.3390/membranes11030179

**Published:** 2021-03-03

**Authors:** Caide Yue, Hongmin Dong, Yongxing Chen, Bin Shang, Yi Wang, Shunli Wang, Zhiping Zhu

**Affiliations:** 1 Institute of Environment and Sustainable Development in Agriculture, Chinese Academy of Agricultural Sciences, Beijing 100081, China; 82101181041@caas.com (C.Y.); chenyongxing@caas.cn (Y.C.); shangbin@caas.cn (B.S.); wangyi01@caas.cn (Y.W.); wangshunli01@caas.cn (S.W.); zhuzhiping@caas.cn (Z.Z.); 2 Key Laboratory of Energy Conservation and Waste Treatment of Agricultural Structures, Ministry of Agriculture and Rural Affairs, Beijing 100081, China

**Keywords:** digestate, ultrafiltration, pore size, membrane flux, membrane fouling, chemical cleaning

## Abstract

Ultrafiltration (UF) can effectively remove large particles, suspended solids, and colloidal substances from anaerobic digestate. However, membrane fouling is a technical challenge in the purification of the digestate by UF. In this study, polyethersulfone (PES) membranes with four pore sizes (50.0, 20.0, 10.0 and 5.0 kDa) were employed to filter anaerobic digestate from swine manure. The effects of temperature, transmembrane pressure (TMP), and cross-flow velocity (CFV) on flux were investigated. The purification effects and fouling characteristics of the four membranes were analyzed. The results revealed that the increase of temperature and CFV can effectively promote UF separation efficiency, but as the TMP exceeded 3.0 bar, the flux increase rates of the four membranes were almost zero. The larger membrane pore size caused the faster flux increase with the increase in pressure. During the batch experiment, the 20.0 kDa membrane showed the lowest flux maintenance ability, while the 5.0 kDa showed the highest ability due to the smaller pore size. All four membranes can effectively remove tetracyclines residues. Elements C, O, and S were the major membrane foulant elements. The dominant bacteria orders of membrane fouling were *Pseudomonadales*, *Xanthomonadales* and *Burkholderiales*. Compared with tap water and citric acid, the membrane cleaning by NaOH and NaClO showed higher flux recovery rates. The 50.0 kDa membrane achieved the best cleaning effects under all cleaning methods.

## 1. Introduction

Anaerobic digestion (AD) can convert organic matters of livestock manure into renewable energy (biomethane) and realize the energy utilization of manure [1,2]. However, the digestate of AD contains high nutrients, suspended solids, and water content, making effective treatment and utilization of digestate difficult to achieve [3]. The solid–liquid separation process is usually used to divide the digestate into the solid and liquid fraction. The solid fraction rich in organic matters and phosphorus can be used to produce organic fertilizer. Meanwhile, the liquid fraction contains high nitrogen and potassium contents, and can be used for nutrient recovery by membrane concentration or struvite production [4]. The conventional solid–liquid separation methods, such as gravity sedimentation, plate filtration and screw extrusion, can only remove large particles [5,6,7], and the liquid fraction still contains large amount of colloids (size of 0.001–1.0 μm) and fine particles [5,6]. Nanofiltration (NF) or reverse osmosis (RO) have been reported to be the effective means for nutrients concentration from the wastewaters/liquid AD effluents [7,8]. However, serious membrane fouling often happens during the liquid phase passing through NF or RO [9]. An effective pretreatment process is urgently needed to alleviate membrane fouling.

Ultrafiltration (UF) is an effective process for the removal of organic particles, colloids, and fine particles of wastewaters, and it has been widely used in tap water purification and municipal wastewater treatment [10,11]. The pore size of UF is between 0.001 and 0.05 μm. Previous studies showed that AD digestate purified by UF retained abundant nutrients such as nitrogen, potassium, and other organic matters in the permeate for the further nutrient recovery [12]. In addition, UF can be used to remove the pathogens existing in digestate, which is beneficial for the microbial contamination control during the farmland use of digestate [11,13]. Therefore, UF is considered as an ideal pretreatment process before NF or RO concentration. Many different types of membranes (inorganic and polymeric) are employed in the UF process, and due to the benefits of relatively low cost, polymeric membranes have been widely used in the UF treatment of wastewaters [9]. However, membrane fouling is one of critical problems encountered in the UF treatment using polymeric membranes, especially when treating high concentration organic wastewater such as liquid AD digestate [14].

UF membrane fouling can be controlled and mitigated from many aspects, such as feed pretreatment [15], membrane module re-design [16], operation parameter optimization [17], process combination [18], and membrane cleaning [19], etc. Many researchers have carried out the studies of UF membrane fouling in treating the livestock wastewaters. For example, Zhan et al. [20] combined paper filtration (50 μm) with polymeric membrane UF (10–100 nm) for the pretreatment of pig farm biogas slurry, and they found that paper filtration can remove macromolecular organics and total suspended solids, which alleviated the membrane fouling of the UF process. Fugère et al. [17] used a tubular UF membrane (polymeric) to filter storage tank manure, fresh manure, sieved manure, and bio-treated manure from different treatment stages. The fresh manure caused severe flux decline, while the other feeds brought the higher membrane flux due to the degradation of floccule, sugars, and proteins during the pretreatment processes. Camilleri et al. [12] also compared the performance of different membrane materials (polysulfone (PS), surface-modified polyvinylidene fluoride (PVDF), and polyethersulfone (PES)) and pointed out that the Zeta potential of the membrane surface may affect the formation of membrane fouling. The PVDF membrane showed better anti-fouling performance than the PS membrane, which was probably due to the higher Zeta potential in the initial stage of filtration. These studies mainly focused on the impact of feedstock pretreatment and membrane materials on the form of UF membrane fouling, but mechanisms of polymeric membrane fouling in treating AD digestate were still not well investigated and needed to further explore.

Membrane fouling might be caused by the difference of fouling characteristics, including complete pore blocking, intermediate pore blocking, cake filtration, and standard pore blocking [21]. Membrane pore size or molecule weight cut off (MWCO) is one of the important parameters that affects the formation process of membrane fouling [22,23]. Generally, comprehensive consideration of factors, including feed characteristics, membrane pore size, membrane flux variations, and membrane fouling composition, are needed for the selection of UF processes [24]. As a result of the high complexity of AD digestate, it is difficult to quantify the characteristics of pollutants (include the size and the morphology of particles) under pressurized condition and select the appropriate membrane pore size. Waeger et al. [25] illustrated the treatment effects and flux variations with different pore sizes of ceramic membranes during the digestate purification process and found that fine particles more easily blocked 200 nm ceramic membrane pores, while UF membranes with lower pore sizes (50 nm and 20.0 kDa) maintained higher membrane flux. Those results cannot be directly applied to polymeric membranes due to their differences in the membrane structure. Therefore, a need for a comprehensive study of the relationships between pore size of polymeric membranes and membrane fouling still remains.

The objectives of this study were to (1) investigate the effects of operating parameters (temperature, transmembrane pressure (TMP), and cross-flow velocity (CFV)) on membrane flux; (2) identify the digestate purification effects of UF membranes; (3) evaluate the impact of pore size on membrane fouling characteristics from three perspectives: flux decline, fouling composition, and membrane cleaning. Specifically, PES membranes with four pore sizes (MWCO: 50.0, 20.0, 10.0, and 5.0 kDa) were employed to filter the AD digestate of swine manure. The influence of membrane pore size on membrane flux, purification effects, and membrane fouling characteristics was addressed. The results of this study could guide the selection of UF membrane pore size and optimize the membrane cleaning strategies in the engineering applications.

## 2. Materials and Methods

### 2.1. Digestate Pretreatment

AD digestate was collected from a swine manure treatment plant located in the Changping district, Beijing, China. This swine farm used a deep–pit system for manure collection. The manure from the piggery houses flowed into a storage tank and was separated by a screw press. After solid–liquid separation, the solid fraction was used for organic fertilizer production, and the liquid fraction was pumped into an anaerobic digester for the biogas production. The digestate samples were taken from effluent of the anaerobic digester. All collected samples were settled for 24 hours in the ambient temperature. The supernatant passed through a 200-mesh sieve (size of 75 μm) and was used as the feedstock of polymer UF membranes.

### 2.2. UF System and Separation Process

**Figure 1:**
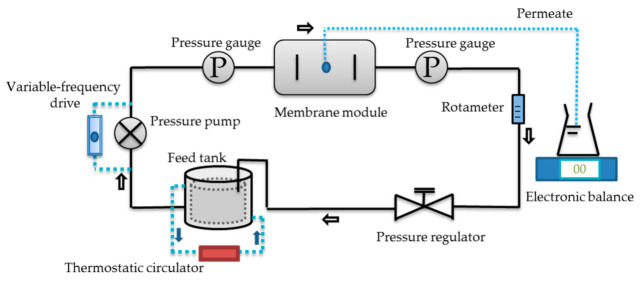
Flow chart of flat-plate cross-flow filtration device.

A flat-plate cross-flow filtration device was used for digestate purification. This device mainly consisted of pressure pump, pressure gauges, feed tank (3 L), membrane module, electronic balance, thermostatic circulator, etc. The size of the flat membrane modules are 130.0, 65.0, and 1.3 mm in length, width, and height, respectively, and its effective membrane area was 84.5 cm^2^. The membranes used in this experiment were provided by RisingSun Membrane technology (Beijing) Co., Ltd. (Beijing, China), and the membrane information is shown in Table 1. Figure 1 shows the flow chart of the device, featuring the digestate from the feed tank pumped into the membrane module. Two pressure gauges were mounted at both sides of the membrane module for transmembrane pressure monitoring. Meanwhile, the permeate flow was measured, and cross-flow velocity (CFV) and temperature were controlled. A volumetric flask (500 mL) and an electronic balance (Max: 800 g; Shanghai Tianmei Balance Instrument Co., Ltd. (Shanghai, China)) were used for the permeate flow monitoring. The CFV was controlled by a variable-frequency drive. Operating temperature was controlled by a thermostatic circulator (DTY-30B; Beijing Detianyou Technology Development Co., Ltd. (Beijing, China)). Before purifying the digestate, the UF membranes were soaked in deionized water for 40 min, and the whole system operated with deionized water as the feedstock until the flux remained stable.

### 2.3. Experimental Design

Four pore size PES membranes (MWCO: 50.0, 20.0, 10.0, and 5.0 kDa) were tested under the same operating parameters. The operating parameters were set to TMP: 1.0, 2.0, 3.0, 4.0, 5.0, 6.0 bar; CFV: 1.0, 1.5, 2.0 m s^−1^; T: 25.0, 30.0, 35.0, 40.0, 45.0 °C, and each operating condition lasted for 5 min to alleviate the influence from membrane fouling. The operating parameters selection referred to the recommendation of equipment manufacturer and previous studies [14,26,27]. The purification of digestate was carried out under batch operating condition, and the flux decline characteristics of UF membranes with different pore sizes were detected. The volume concentration factor was set to 3. Scanning electron microscope-energy spectroscopy (SEM-EDS) was used to analyze the morphology and composition of fouled membranes. The bacterial microbial community of membrane fouling was analyzed by a 16s rDNA high-throughput sequencing method. At end of the batch experiment, membrane cleaning (30 min) was carried out. The cleaning effects of different chemical agents (Sodium hydroxide (NaOH, 1 wt ‰), citric acid (1 wt ‰) and sodium hypochlorite (NaClO, 1 wt ‰)) were evaluated. The operating conditions of membrane cleaning were set to TMP, 1.0 bar; T, 25.0 °C; CFV, 1.5 m s^−1^.

### 2.4. Sampling and Analysis

The influent, concentrate, and permeate of each batch of test were sampled, and the sampling volume was 250 mL. Those samples were immediately stored under 4.0 °C condition and tested in a timely manner. The methods and instruments for COD, NH_3_-N, TN, K, and TP tests were described in detail in our previous study [7]. The absorbance at 254 nm (UV_254_) was measured by a UV Spectrophotometer (Shimadzu UV-2450, Tokyo, Japan). A three-dimensional excitation emission matrix (3D-EEM) was assayed by a Hitachi F-4700 fluorescence spectrophotometer. Before the UV_254_ and fluorescence measurements, the samples were filtered with a 0.45 μm filter membrane and diluted 125 times to meet the measurement range. Samples used for antibiotics detection were stored under −80 °C condition, and measured by high-performance liquid chromatography- mass spectrometer. Thirty-three kinds of antibiotics commonly used on livestock have been tested as before [28]. The membrane morphology and fouling structure were observed and analyzed using SEM-EDS (Hitachi S-4800, Tokyo, Japan). The membrane cross-section specimens were prepared via the cryogenic fracture method followed by gold sputtering. The membrane samples used for microbial community analysis were stored under −80 °C condition and then transported to Shanghai Majorbio Bio-pharm Technology Co., Ltd. (Shanghai, China). The V4-V5 region of the extracted DNA was amplified by PCR reactions with 515F(5′-GTGCCAGCMGCCGCGG-3′) and 907R(5′-CCGTCAATTCMTTTRAGTTT-3′) primer pairs. The data were analyzed by Majorbio Cloud Platform.

### 2.5. Membrane Performance

The membrane flux (*J*) was defined as the volume of liquid passing through per unit surface area per unit time, (L m^−2^ h^−1^):

(1) $$J=\frac{V}{T\times A}$$

where *V* is the volume of the permeate, *L*; *A* is the membrane area, m^2^; and *T* is the filtration time, h.

The membrane flux recovery rate (FRR) was calculated by comparing the pure water flux before filtration and after cleaning, (%):

(2) $$FRR=\frac{J}{J_{0}}\times 100$$

where *J*_0_ is the pure water flux before filtration, L m^−2^ h^−1^; and *J* is the pure water flux after cleaning, L m^−2^ h^−1^.

### 2.6. Statistical Analysis

Experimental data were recorded and calculated using Microsoft Excel 2010. Plotting and data analysis were obtained by Sigma plot software (Version 12.5, Systat Software, Inc., San Jose, CA, USA). Origin 2018 was used for the constructing of EDS and 3D-EEM images. Statistical analysis was performed using IBM SPSS Statistics 20.0 for Windows. One-way ANOVA was used to determine the significant differences (*p* < 0.05) among groups.

## 3. Results and Discussion

### 3.1. Effects of Operating Parameters on Membrane Flux

#### 3.1.1. Operating Temperature

**Figure 2:**
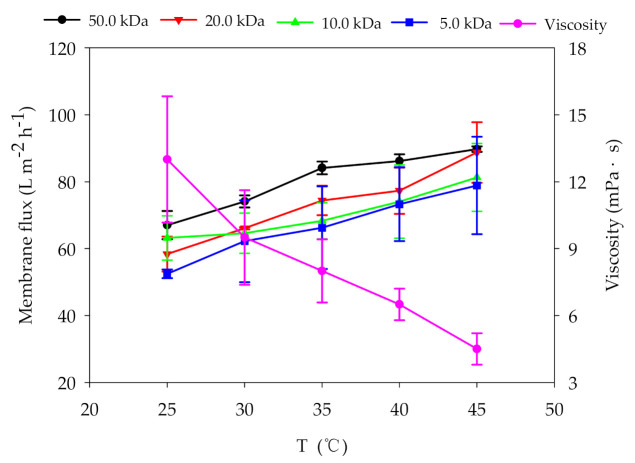
Effects of temperature on viscosity of digestate and flux of ultrafiltration (UF) membranes (TMP: 3.0 bar; CFV: 1.5 m s^−1^).

Increasing the operating temperature can improve the rheological properties of digestate. As shown in Figure 2, the viscosity of the digestate dropped from 13.0 ± 2.8 to 4.5 ± 0.7 mPa·s, with the increasing of operating temperature from 25.0 °C to 45.0 °C. At the same time, the membrane fluxes of 50.0, 20.0, 10.0 and 5.0 kDa membranes increased by 34.2%, 52.1%, 28.6%, and 50.3%. This result indicated that temperature increasing improved the permeability of UF membrane. The changes in rheological properties of digestate might cause by the denaturation of microbial metabolites such as proteins and polysaccharides. Liu et al. [29] studied the influence of temperature on the physical–chemical properties of the digestate from pig farm and found that higher temperature can increase the movement intensity of particles and destroy the stable state of organic polymer compounds (such as soluble carbohydrates, soluble proteins, organic acids, etc.). However, the effects of the increasing temperature on subsequent membrane fouling are still controversial. Alresheedi et al. [26] studied the membrane fouling characteristics during UF for drinking water production and pointed out that UF separation under low temperature condition accelerated the deposition of pollutants in membrane pores and aggravated irreversible membrane fouling. Ng’s [27] research reported that compared with lower temperature, the UF filtration of skimmed milk under 50.0 °C caused irreversible membrane fouling by pore expansion. The above results indicated that the formation of UF membrane fouling was affected by multiple variables, which are not only related to operating parameters but also dependent on feed characteristics. Therefore, it is necessary to further evaluate the influence of temperature on membrane fouling during digestate purification to avoid the aggravation of irreversible fouling.

#### 3.1.2. Transmembrane Pressure

**Figure 3:**
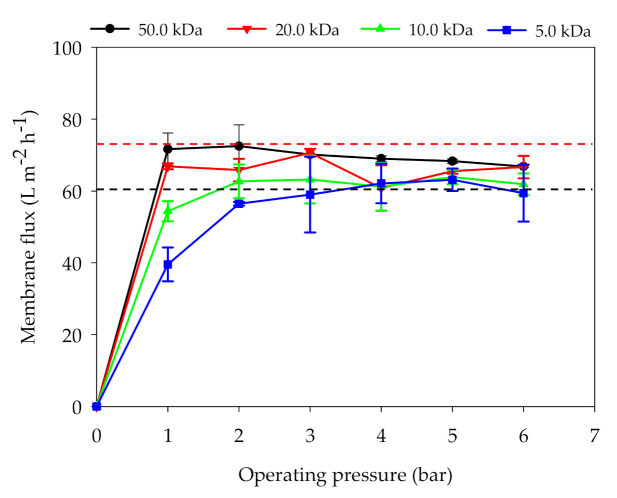
Effects of TMP on the flux of UF membranes with different pore sizes (T: 25.0 °C; CFV: 1.5 m s^−1^).

As presented in Figure 3, the flux increase rates of the four membranes can be arranged in the start-up stage (TMP < 3.0 bar) in the series that follows: 50.0 kDa > 20.0 kDa > 10.0 kDa > 5.0 kDa. It was revealed that the larger membrane pore size caused the faster flux increase rate with the increase in pressure. However, there is a mutually restrictive relationship between membrane flux and permeate quality using membrane purification. Under the same operating pressure, the UF membrane with a smaller pore size encountered a higher membrane inherent resistance in the digestate purification process, resulting in a higher permeate quality [25]. At 1.0 bar, the fluxes of 50.0 and 20.0 kDa membranes had already reached 65 L m^−2^ h^−1^ as the highest. However, the fluxes of 10.0 kDa and 5.0 kDa UF membranes were still rising at the same TMP. At 3.0 bar, all four UF membranes can reach to 60.0 L m^−2^ h^−1^ approximately. However, as the TMP exceeded 3.0 bar, the flux increase rates of the four membranes were almost zero. This phenomenon indicated that the TMP of the UF membrane should be lower than 3.0 bar during digestate purification. The reasons for this phenomenon may be that compressible organic aggregates (such as colloids and particles) in digestate accumulated on the membrane surface or penetrate into membrane pores under the higher TMP, resulting in serious membrane fouling. Magdalena et al. [30] found that an increase in TMP caused an increase in the total hydraulic resistance of membrane, resulting in more solid pollutants accumulating on the membrane surface and blocking the pores. Concentration polarization was also assumed to have an effect on the UF separation process, as the digestate contained a large amount of salts (EC = 7.7 ± 0.05 ms cm^−1^). Alexandre et al. [31] pointed out that the concentration polarization is more intense at higher TMP, leading to the formation of a more selective layer onto the membrane surface. 

#### 3.1.3. Cross-Flow Velocity

**Figure 4:**
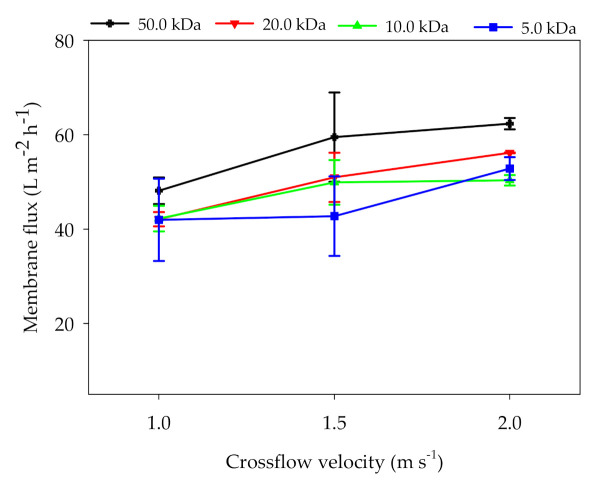
Effects of CFV on the flux of UF membranes with different pore sizes (TMP: 3.0 bar; T: 25.0 °C).

Figure 4 shows the trends of membrane flux changes under different CFVs. With the increase of CFV, the fluxes of the four membranes increased. Compared with 1.0 m s^−1^, the membrane fluxes at CFV 2.0 m s^−1^ were increased by 29.5%, 33.4%, 19.2%, and 26.0%, respectively. Guo et al. [14] also found the membrane flux can be improved by the increase of CFV when purifying the digestate with tubular membrane. However, the relationship between membrane pore size and CFV was not found in our experiment. Hwang et al. [32] found that the effects of CFV on membrane flux were more significant under lower TMP or for the membrane with a larger pore size. Previous studies illustrated that the increase of CFV can improve the hydrodynamic conditions and increase the shear force between liquid and membrane, which can effectively reduce the concentration polarization, particles deposition, and organic polymer adhesion [31,33]. Shear force enhancement on the membrane surface can induce turbulence and accelerate the diffusion of deposited ions on the membrane surface to the bulk stream [34]. The high CFV can enhance mass transfer phenomena and mitigate membrane fouling, but this leads to high operation costs and limits the economical application. In order to reduce the CFV requirement, Tobias et al. [35] used ozone to pretreat anaerobic digestate, and they found that UF flux could be improved by 50–80% by ozonation. Ozone treatment reduced the biopolymer concentration and apparent viscosity.

#### 3.1.4. Membrane Flux Variation

**Figure 5:**
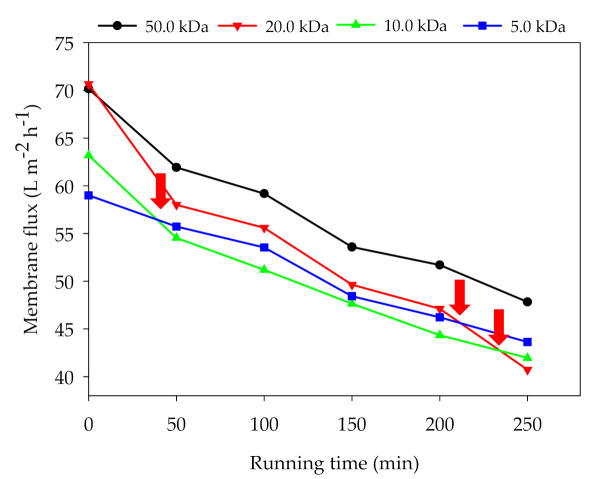
Effect of pore size on membrane flux decline (TMP: 3.0 bar; T: 25.0 °C).

For the batch experiment, the fluxes of all membranes declined, with the gradual increase of feed concentration and the intensification of membrane fouling (Figure 5). After 40 min running, the flux of the 10.0 kDa membrane was lower than that of the 5.0 kDa membrane. At about 210 min, the flux of the 20.0 kDa membrane was also lower than that of the 5.0 kDa membrane. After 250 min running, the flux of the 50.0, 20.0, 10.0, and 5.0 kDa membranes were decreased by 31.8%, 42.4%, 336 %, and 26.1%, respectively. It was interesting that the 20.0 kDa membrane showed the worst flux maintenance ability. The membrane flux maintenance ability not only depends on the nature of the membrane material but also on the feed characteristics (particle size distribution, organic matter concentration) and operating parameters. Waeger et al. [25] found that membrane pores with a larger diameter are more susceptible to internal clogging through smaller particles. In this study, we assumed that the 20.0 kDa membrane was more likely to be blocked by particles than the 10.0/5.0 kDa membranes. The 50.0 kDa membrane maintained the highest membrane flux throughout the operating cycle, probably because the fine particles in the digestate can pass through larger membrane pores. The above results elucidated that the pore size of UF membrane does affect the changes of membrane flux and the formation of membrane fouling.

### 3.2. Purification Effect of UF on Digestate

#### 3.2.1. Changes in Physicochemical Characteristics

Size exclusion is the major mechanism of rejection in UF, and the removal efficiency of UF on impurity in digestate may be impacted by its chemical speciation and distribution [30]. Table 2 shows the changes of physicochemical characteristics of the influent, concentrates, and permeates. The COD concentration in the permeate of 50.0, 20.0, 10.0, and 5.0 kDa membranes decreased significantly (*p* < 0.05), with the removal rates of 78.4%, 86.9%, 88.5%, and 89.6%, respectively. This observation indicated that membranes with smaller pore sizes were beneficial to the COD removal. The COD concentration in 50.0 kDa permeate was the highest, 290.0 ± 70.0 mg L^−1^, and that of other permeates were lower than 240 mg L^−1^, which meets the requirement of COD discharge standard in China [36]. The COD removal rates obtained in this study were higher than those in Carlos’s study [37]. For TP, more than 70% removal efficiency was achieved for all four membranes, indicating that TP in digestate mainly existed in granular or colloidal form. As for the TP concentration of different membranes permeates, no significant differences were found. Masse et al. [38] found that only 20% of the TP was soluble, and 50% of the TP was associated with particles (0.45–10 μm) in digested swine manure. Since the major part of the nitrogen in digestate is mainly water-soluble NH_3_-N, the elimination of nitrogen compounds was rather low for UF membranes. Approximately 80% of the TN entered the permeate, and the rest of the nitrogen was trapped in the concentrate or volatilized into atmosphere. In addition, most of K in digestate is also water-soluble, so there was almost no loss after UF. The above results once again proved that UF can effectively retain nitrogen and potassium in permeate and provide preconditions for the nutrients concentration by NF or RO.

#### 3.2.2. Antibiotics Removal

In order to clarify the distribution of antibiotics in digestate before and after UF purification, 33 kinds of antibiotics were analyzed in the influent, concentrates, and permeates. The results showed that only four tetracycline antibiotics were detected in the influent, namely oxytetracycline (OTC) 89.1 ± 33.8 μg L^−1^, chlorotetracycline (CTC) 1.4 ± 0.1 μg L^−1^, tetracycline (TET) 1.4 ± 0.1 μg L^−1^, and doxycycline (DOTC) 2.2 ± 0.1 μg L^−1^. These antibiotics have been commonly used as veterinary drugs to prevent and treat animal diseases [39]. Theoretically, UF membranes do not have the ability to retain antibiotics with a molecular weight less than 1.0 kDa in pure aqueous solutions [40]. However, it was found that the UF membranes removed most antibiotics from digestate in this study. Except for a small amount of OTC that was detected in 50.0 kDa membrane permeate, no antibiotics were detected in other membrane permeates. According to previous results, it indicated that antibiotics in digestate adsorbed on the solid phase. Xu et al. [41] investigated the removal of antibiotics by a sequencing-batch membrane bioreactor for swine wastewater treatment and found that tetracyclines can be adsorbed on the solid phase. Xu et al. [42] found that extracellular polymeric substances (EPS) in activated sludge from urban sewage treatment plants captured tetracycline molecules mainly through the π–π stacking reaction of the benzene rings of TC and EPS proteins. Further clarifying the distribution of antibiotics in different particulates and colloidal substances in digestate is essential so as to better explain the interception effect of antibiotics by the physical screening methods such as membrane separation and mechanical separation.

#### 3.2.3. Dissolved Organic Matter

**Figure 6:**
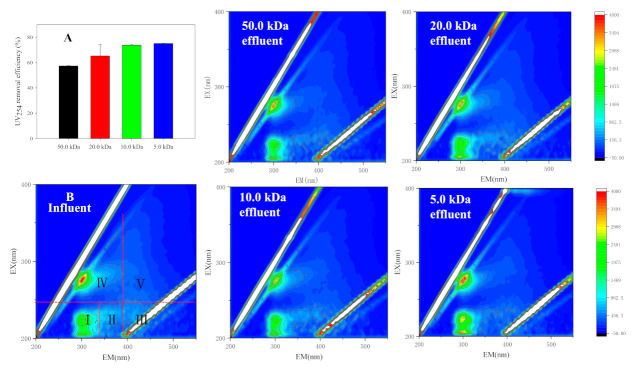
(**A**) UV_254_ removal rates of UF membranes with different pore size and (**B**) three-dimensional excitation emission matrix (3D-EEM) profiles.

UV_254_ is a parameter to reflect the aromaticity or organic matters with double bounds [43]. As shown in Figure 6A, the UV_254_ removal rates of the four membranes were all above 50%. In addition, the UV_254_ removal efficiency was highly related to the membrane pore size. 3D-EEM analysis was used to characterize the variation of fluorescent organic substances in digestate, and the analysis results are shown in Figure 6B. The soluble organic fluorescent substances that are commonly recognized could be divided into five regions, including simple aromatic proteins I (region_I), aromatic proteins Ⅱ (region_II), fulvic acid-like substances (region_Ⅲ), soluble microbial by-product-like substances (SMBP, region_Ⅳ), and humic acid-like substances (region_Ⅴ). Table 3 shows the integral standard volume of the fluorescence region before and after UF purification. The results showed that the main soluble fluorescent organic substances were aromatic proteinsI and SMBP. Although part of the fluorescent substances in digested slurry can be retained by UF membrane, most of the fluorescent substances passed through UF membranes. Permeable fluorescent substances such as humic acid and fulvic acid can be concentrated and recovered by subsequent NF or RO membranes, which is beneficial to improve the quality of organic liquid fertilizer.

### 3.3. Membrane Fouling Characteristics

#### 3.3.1. SEM-EDS

**Figure 7:**
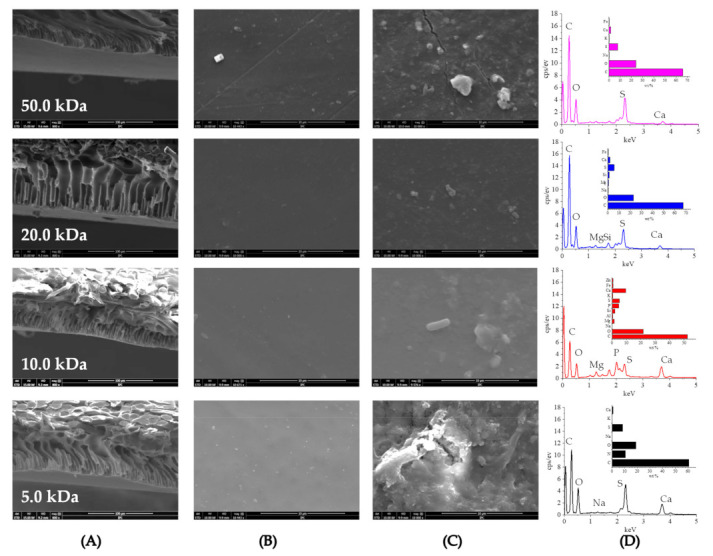
SEM images of (**A**) cross-sections of UF membranes (×800), (**B**) virgin membrane surface (×10,000), (**C**) fouled membrane surface (×10,000), (**D**) energy-dispersive spectroscopy (EDS) of fouling.

As seen in Figure 7A, the cross-section of the PES UF membranes used in this study is an asymmetric structure, and the support layer is a finger-like structure. Comparing the morphology of the membrane surface before and after fouling, the fouled membrane surface was covered by suspended particles, colloidal substances, and microorganisms, which forming a thick cake layer (Figure 7B,C). The cake layer contains complex polymer mixtures such as EPS and extracellular polymeric substances (SMP) produced by microbial metabolism [42,44]. The main components of EPS and SMP were protein, polysaccharides, and humus, and these organic matters can be adsorbed on the membrane surface, resulting in serious membrane fouling [45,46]. EDS analysis found that the organic elements of the foulant mainly include C, O, and S, while the inorganic elements mainly include Ca, Na, Mg, and K (Figure 7D). The proportions of elements in the pollutants showed that organic matters were the main components of membrane fouling. During the pressure filtration process, the deformable organic aggregates were gradually settled and compacted on the membrane surface.

#### 3.3.2. Bacterial Community Analysis

**Figure 8:**
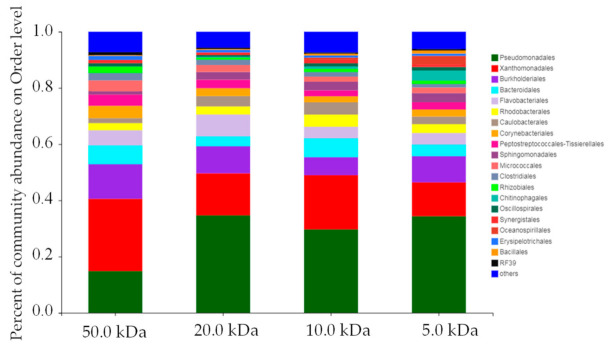
Relative abundance of bacterial communities of membrane fouling.

The relative abundance of the bacterial community of membrane fouling showed that more than 20 kinds of bacteria were detected on the order level (Figure 8). Among them, *Pseudomonadales*, *Xanthomonadales*, *Burkholderiales*, *Bacteroidales*, and *Flavobacteriales* were the dominant bacteria. The 50.0 kDa membrane was mainly polluted by *Xanthomonadales*; the other three membranes were mainly polluted by *Pseudomonadales*. The adsorption capacity of bacteria on the membrane surface is mainly related to membrane properties (surface electrical properties, hydrophilicity and hydrophobicity, etc.) and the characteristics of bacteria (size, metabolic type) [47,48]. It should be pointed out that bacteria attached to the surface of membrane may multiply and form biofilm. Therefore, it is necessary to control the bacterial contamination during digestate purification.

#### 3.3.3. Membrane Cleaning

**Figure 9:**
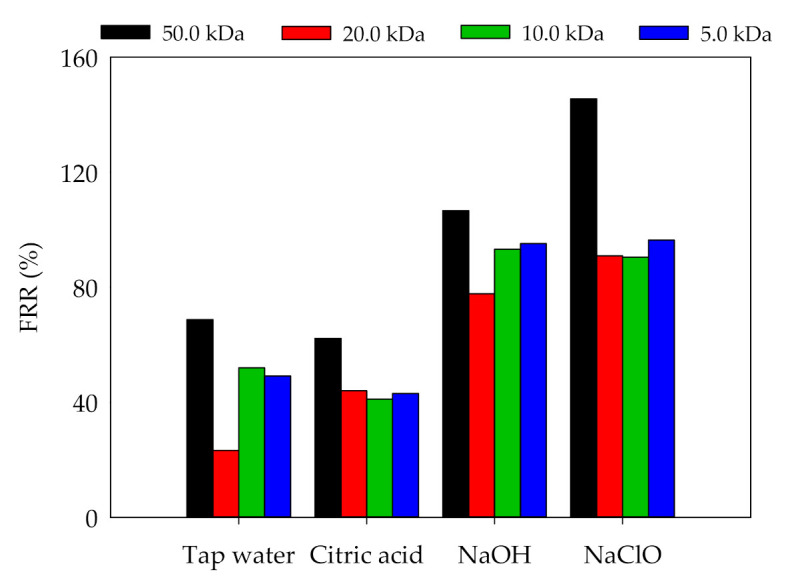
Flux recovery rates (FRR_S_) of UF membranes with different cleaning agents.

**Figure 10:**
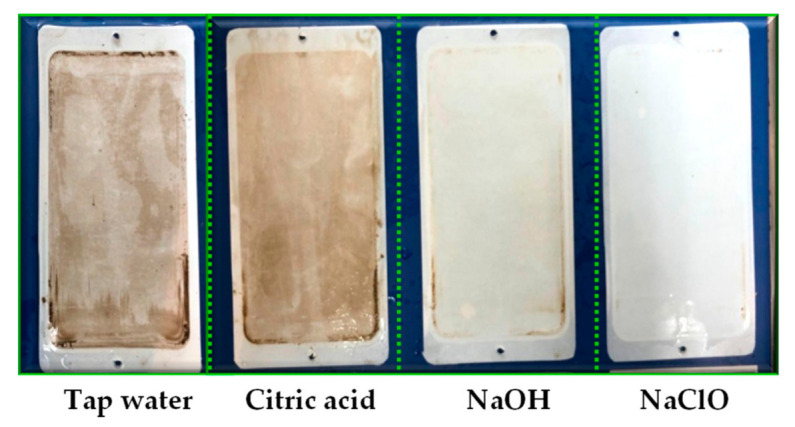
Cleaning effects of the 50.0 kDa membranes with different cleaning agents.

Membrane cleaning is one of the important procedures to maintain the high membrane flux. Membrane cleaning agents should be selected according to the type of pollutions. Four cleaning agents, namely tap water, citric acid, NaOH, and NaClO were selected for membrane cleaning (Figure 9). The results showed that (1) the tap water cleaning method is based on hydraulic flushing and dissolution. The FRRs after tap water cleaning were limited (FRRs < 70%) (Figure 10). Among the four membranes, 20.0 kDa membrane had the lowest FRR, indicating that it was easier to form membrane pore clogging. (2) Citric acid is used to clean inorganic pollutants of membrane fouling through dissolution and chelation. The FRRs after citric acid cleaning were less than 60%. (3) NaOH solution can hydrolyze and dissolve organic pollutants on the membrane, and the cleaning effects obtained by NaOH cleaning were better than those obtained by citric acid. (4) NaClO can oxidize and remove pollutants on the membrane surface or pores. The FRRs of 50.0, 20.0, 10.0, and 5.0 kDa membranes after NaClO cleaning were 145.5%, 90.9%, 90.5%, and 96.5%, respectively. The pore size of membrane also showed effects on FRR, and the 50.0 kDa membrane obtained higher FRRs under four cleaning methods, indicating that the larger membrane pore size were associated with the higher FRR.

## 4. Conclusions

The increase of temperature and CFV can effectively promote UF separation efficiency, but as the TMP exceeded 3.0 bar, the flux increase rates of the four membranes were almost zero. The larger membrane pore size caused the faster flux increase with the increase in pressure. During the batch experiment, the 20.0 kDa membrane showed the lowest flux maintenance ability, while the 5.0 kDa showed the highest ability due to the smaller pore size. All four membranes can effectively remove tetracyclines residues. Elements of C, O, and S were the major membrane foulant elements. The dominant bacteria orders of membrane fouling were *Pseudomonadales*, *Xanthomonadales*, and *Burkholderiales*. Compared with tap water and citric acid, the membrane cleaning by NaOH and NaClO showed higher flux recovery rates. The 50.0 kDa membrane achieved the best cleaning effects under all cleaning methods.

## Figures and Tables

**Table 1:** Characteristics of commercial membranes used in this study.

Membrane Type	MWCO (Da)	Contact Angle (°)	Pure Water Flux (L m^−2^ h^−1^) ^1^
PES 5.0	5000	74.1 ± 6.9	151.4 ± 16.3
PES 10.0	10,000	64.4 ± 1.7	203.8 ± 9.4
PES 20.0	20,000	52.0 ± 4.8	309.6 ± 19.4
PES 50.0	50,000	68.7 ± 2.2	593.6 ± 84.5

^1^ Test condition: TMP = 3.0 bar; T = 25.0 °C; CFV = 1.5 m s^−1^.

**Table 2:** Physicochemical characteristics of the influent, concentrates, and permeates ^1^.

Treatment	Types	COD/ (mg⋅L^−1^)	TP/ (mg⋅L^−1^)	NH_3_-N/ (mg⋅L^−1^)	TN/ (mg⋅L^−1^)	K/ (mg⋅L^−1^)	OTC/ (μg L^−1^)	CTC/ (μg L^−1^)	TET/ (μg L^−1^)	DOTC/ (μg L^−1^)
	Influent	1345 ± 261.7 b	100.6 ± 20.0 ab	647.5 ± 31.8 a	660 ± 254.6 a	1092.8 ± 50.6 a	89.1 ± 33.8	1.4 ± 0.1	24.4 ± 9.6 b	2.2 ± 0.1
50.0 kDa	Concentrate ^2^	2500 ± 1244.5 ab	52.7 ± 69.6 ab	597.5 ± 17.7 ab	800 ± 141.4 a	1106 ± 58.7 a	156.1 ± 132.4	6.4 ± 0.1	62.0 ± 0.1	13.7 ± 0.1
	Permeate	290 ± 70.7 c	25.6 ± 0.87 b	517.5 ± 31.8 b	530 ± 240.4 a	1081.8 ± 10.3 a	3.0 ± 0.1	- ^3^	-	-
20.0 kDa	Concentrate	3470 ± 98.9 a	189.2 ± 14.8 a	610.0 ± 21.2 ab	830 ± 42.4 a	1148.3 ± 63.9 a	247.7 ± 2.08 c	4.4 ± 0.06	70.7 ± 7.9	12.6 ± 0.8
	Permeate	175 ± 35.4 c	30.5 ± 0.9 b	625.0 ± 28.3b	530 ± 183.8 a	1087.3 ± 72.5 a	-	-	-	-
10.0 kDa	Concentrate	3410 ± 240.4 a	176.9 ± 28.7 a	645.0 ± 7.1 a	720 ± 226.3 a	1094.8 ± 107.1 a	283.2 ± 56.7	6.4 ± 2.7	84.7 ± 15.5	14.2 ± 5.2
	Permeate	155.0 ± 21.2 c	29.3 ± 2.6 b	537.5 ± 53.0 b	490 ± 14.1 a	1053.3 ± 43.5 a	-	-	-	-
5.0 kDa	Concentrate	2680.0 ± 551.5 ab	190.5 ± 82.6 ab	620.0 ± 14.1 ab	810 ± 14.1 a	1120.3 ± 63.9 a	241.0 ± 12.4	3.5 ± 0.1	57.6 ± 14.4	8.5 ± 2.7
	Permeate	140.0 ± 0.01 c	29.3 ± 1.7 b	572.5 ± 24.7 ab	510 ± 127.3 a	1131 ± 105.4 a	-	-	-	-

^1^ Values within columns followed by the same letter are not significantly different at *p* < 0.05; ^2^ Volume concentration factor was set to 3; ^3^ “-“ means lower than the detection limit.

**Table 3:** The integral standard volume of the fluorescence region before and after UF purification.

Region	Organic	Ex(nm)	Em(nm)	Integral Standard Volume (au·nm^2^)				
				Influent	50.0 kDa Effluent	20.0 kDa Effluent	10.0 kDa Effluent	5.0 kDa Effluent
I	Aromatic proteins I	200~250	280–330	117,160	95,434	109,728	100,691	125,757
II	Aromatic proteins II	200~250	330–380	59,069	39,427	43,380	44,546	39,970
Ⅲ	Fulvic acid-like	200~250	380–550	381,601	285,436	283,485	276,636	357,423
Ⅳ	SMBP	250~340	280–380	492,550	387,255	356,757	357,817	357,841
Ⅴ	Humic acid-like	340~400	380–550	386,489	234,302	250,661	200,942	243,290

## Data Availability

Data are contained within the article.

## Nomenclature

AD	anaerobic digestion
CTC	chlorotetracycline
CFV	cross-flow velocity (m s^−1^)
DOTC	doxycycline
EPS	extracellular polymeric substances
FRR	membrane flux recovery rate (%)
J	membrane flux (L m^−2^ h^−1^)
MWCO	molecule weight cut off
NF	nanofiltration
UF	ultrafiltration
OTC	oxytetracycline
PES	polyethersulfone
RO	reverse osmosis
SEM-EDS	scanning electron microscope-energy spectroscopy
SMP	soluble microbial products
TET	tetracycline
TMP	transmembrane pressure
3D-EEM	three-dimensional excitation emission matrix

## References

[1] Dai X., Gai X., Dong B. (2014). Rheology evolution of sludge through high-solid anaerobic digestion. Bioresour. Technol..

[2] Tao Y., Ersahin M.E., Ghasimi D.S., Ozgun H., Wang H., Zhang X., Guo M., Yang Y., Stuckey D.C., Van Lier J.B. (2020). Biogas productivity of anaerobic digestion process is governed by a core bacterial microbiota. Chem. Eng. J..

[3] Zhang W., Wang H., Li L., Li D., Wang Q., Xu Q., Wang D. (2019). Impact of molecular structure and charge property of chitosan based polymers on flocculation conditioning of advanced anaerobically digested sludge for dewaterability improvement. Sci. Total. Environ..

[4] Guilayn F., Jimenez J., Rouez M., Crest M., Patureau D. (2019). Digestate mechanical separation: Efficiency profiles based on anaerobic digestion feedstock and equipment choice. Bioresour. Technol..

[5] Masse L., Massé D.I., Beauséjour R. (2010). Effects of Polymer Charge Density and Molecular Weight on Flocculation Treatment of Swine Manure at Various Dry Matter Contents. Trans. ASABE.

[6] Burton C. (2007). The potential contribution of separation technologies to the management of livestock manure. Livest. Sci..

[7] Yue C., Dong H., Zhang W., Zhu Z., Yin F., Wang S. (2020). Effects of Membrane Concentration Processes on Flux, Nutrient Recovery, and Antibiotic Isolation for Anaerobically Digested Slurry from Swine Manure. Trans. ASABE.

[8] Han Z., Wang L., Duan L., Zhu S., Ye Z., Yu H. (2015). The electrocoagulation pretreatment of biogas digestion slurry from swine farm prior to nanofiltration concentration. Sep. Purif. Technol..

[9] Masse L., Massé D., Pellerin Y. (2007). The use of membranes for the treatment of manure: A critical literature review. Biosyst. Eng..

[10] Sanchez A.A., Mladenov N., Wasswa J. (2020). Fluorescent compounds retained by ultrafiltration membranes for water reuse. J. Membr. Sci..

[11] Lee S., Ihara M., Yamashita N., Tanaka H. (2017). Improvement of virus removal by pilot-scale coagulation-ultrafiltration process for wastewater reclamation: Effect of optimization of pH in secondary effluent. Water Res..

[12] Camilleri-Rumbau M., Norddahl B., Wei J., Christensen K., Søtoft L.F. (2014). Microfiltration and ultrafiltration as a post-treatment of biogas plant digestates for producing concentrated fertilizers. Desalination Water Treat..

[13] Silkina A., Zacharof M.-P., Hery G., Nouvel T., Lovitt R.W. (2017). Formulation and utilisation of spent anaerobic digestate fluids for the growth and product formation of single cell algal cultures in heterotrophic and autotrophic conditions. Bioresour. Technol..

[14] Guo X., Jin X. (2013). Treatment of Anaerobically Digested Cattle Manure Wastewater by Tubular Ultrafiltration Membrane. Sep. Sci. Technol..

[15] Ma B., Xue W., Li W., Hu C., Liu H., Qu J. (2018). Integrated Fe-based floc-membrane process for alleviating ultrafiltration membrane fouling by humic acid and reservoir water. J. Membr. Sci..

[16] Li G.-P., Zhang L.-Z. (2017). Conjugate heat and mass transfer in a cross-flow hollow fiber membrane bundle used for seawater desalination considering air side turbulence. J. Membr. Sci..

[17] Fugere R., Mameri N., Gallot J., Comeau Y. (2005). Treatment of pig farm effluents by ultrafiltration. J. Membr. Sci..

[18] Ruan H., Yang Z., Lin J., Shen J., Ji J., Gao C., Van Der Bruggen B. (2015). Biogas slurry concentration hybrid membrane process: Pilot-testing and RO membrane cleaning. Desalination.

[19] Goh P., Lau W., Othman M., Ismail A. (2018). Membrane fouling in desalination and its mitigation strategies. Desalination.

[20] Zhan Y., Yin F., Yue C., Zhu J., Zhu Z., Zou M., Dong H. (2020). Effect of Pretreatment on Hydraulic Performance of the Integrated Membrane Process for Concentrating Nutrient in Biogas Digestate from Swine Manure. Membranes.

[21] Kirschner A.Y., Cheng Y.-H., Paul D.R., Field R.W., Freeman B.D. (2019). Fouling mechanisms in constant flux crossflow ultrafiltration. J. Membr. Sci..

[22] Wang L., Wang X. (2006). Study of membrane morphology by microscopic image analysis and membrane structure parameter model. J. Membr. Sci..

[23] Maaz M., Yasin M., Aslam M., Kumar G., Atabani A., Idrees M., Anjum F., Jamil F., Ahmad R., Khan A.L. (2019). Anaerobic membrane bioreactors for wastewater treatment: Novel configurations, fouling control and energy considerations. Bioresour. Technol..

[24] Belfort G., Davis R.H., Zydney A.L. (1994). The behavior of suspensions and macromolecular solutions in crossflow microfiltration. J. Membr. Sci..

[25] Waeger F., Delhaye T., Fuchs W. (2010). The use of ceramic microfiltration and ultrafiltration membranes for particle removal from anaerobic digester effluents. Sep. Purif. Technol..

[26] Alresheedi M.T., Basu O.D. (2020). Interplay of water temperature and fouling during ceramic ultrafiltration for drinking water production. J. Environ. Chem. Eng..

[27] Ng K.S., Dunstan D.E., Martin G.J. (2018). Influence of processing temperature on flux decline during skim milk ultrafiltration. Sep. Purif. Technol..

[28] Yin F., Lin S., Zhou X., Dong H., Zhan Y. (2021). Fate of antibiotics during membrane separation followed by physical-chemical treatment processes. Sci. Total. Environ..

[29] Liu G.-J., Liu Y., Wang Z.-Y., Lei Y.-H., Chen Z.-A., Deng L.-W. (2015). The effects of temperature, organic matter and time-dependency on rheological properties of dry anaerobic digested swine manure. Waste Manag..

[30] Zielińska M., Bernat K., Mikucka W. (2020). Membrane Bioreactor Technology: The Effect of Membrane Filtration on Biogas Potential of the Excess Sludge. Membranes.

[31] Giacobbo A., Bernardes A.M., Rosa M.J.F., De Pinho M.N. (2018). Concentration Polarization in Ultrafiltration/Nanofiltration for the Recovery of Polyphenols from Winery Wastewaters. Membranes.

[32] Hwang K.-J., Sz P.-Y. (2011). Effect of membrane pore size on the performance of cross-flow microfiltration of BSA/dextran mixtures. J. Membr. Sci..

[33] Hartinger M., Schiffer S., Heidebrecht H.-J., Dumpler J., Kulozik U. (2020). Milk protein fractionation by custom-made prototypes of spiral-wound microfiltration membranes operated at extreme crossflow velocities. J. Membr. Sci..

[34] Chen G., Yang X., Lu Y., Wang R., Fane A.G. (2014). Heat transfer intensification and scaling mitigation in bubbling-enhanced membrane distillation for brine concentration. J. Membr. Sci..

[35] Gienau T., Ehrmanntraut A., Kraume M., Rosenberger S. (2020). Influence of Ozone Treatment on Ultrafiltration Performance and Nutrient Flow in a Membrane Based Nutrient Recovery Process from Anaerobic Digestate. Membranes.

[36] Ministry of Ecology and Environment of the People’s Republic of China Discharge Standard of Pollutants for Livestock and Poultry Breeding. http://www.mee.gov.cn/ywgz/fgbz/bz/bzwb/shjbh/swrwpfbz/200301/t20030101_66550.shtml.

[37] Carbonell-Alcaina C., Soler-Cabezas J.L., Bes-Piá A., Vincent-Vela M.C., Mendoza-Roca J.A., Pastor-Alcañiz L., Álvarez-Blanco S. (2020). Integrated Membrane Process for the Treatment and Reuse of Residual Table Olive Fermentation Brine and Anaerobically Digested Sludge Centrate. Membranes.

[38] Masse L., Massé D.I., Beaudette V., Muir M. (2005). Size distribution and composition of particles in raw and anaerobically digested swine manure. Trans. ASAE.

[39] Gros M., Marti E., Balcázar J.L., Boy-Roura M., Busquets A., Colón J., Sànchez-Melsió A., Lekunberri I., Borrego C.M., Ponsá S. (2019). Fate of pharmaceuticals and antibiotic resistance genes in a full-scale on-farm livestock waste treatment plant. J. Hazard. Mater..

[40] Kümmerer K. (2009). Antibiotics in the aquatic environment—A review—Part, I. Chemosphere.

[41] Xu Z., Song X., Li Y., Li G., Luo W. (2019). Removal of antibiotics by sequencing-batch membrane bioreactor for swine wastewater treatment. Sci. Total Environ..

[42] Xu Q., Han B., Wang H., Wang Q., Zhang W., Wang D. (2020). Effect of extracellular polymer substances on the tetracycline removal during coagulation process. Bioresour. Technol..

[43] Altmann J., Massa L., Sperlich A., Gnirss R., Jekel M. (2016). UV254 absorbance as real-time monitoring and control parameter for micropollutant removal in advanced wastewater treatment with powdered activated carbon. Water Res..

[44] Liang S., Liu C., Song L. (2007). Soluble microbial products in membrane bioreactor operation: Behaviors, characteristics, and fouling potential. Water Res..

[45] Fane A., Fell C., Suki A. (1983). The effect of ph and ionic environment on the ultrafiltration of protein solutions with retentive membranes. J. Membr. Sci..

[46] Rongwong W., Goh K., Sethunga D., Bae T.-H. (2019). Fouling formation in membrane contactors for methane recovery from anaerobic effluents. J. Membr. Sci..

[47] Ishizaki S., Fukushima T., Ishii S., Okabe S. (2016). Membrane fouling potentials and cellular properties of bacteria isolated from fouled membranes in a MBR treating municipal wastewater. Water Res..

[48] Li Z., Lin L., Liu X., Wan C., Lee D.-J. (2020). Understanding the role of extracellular polymeric substances in the rheological properties of aerobic granular sludge. Sci. Total. Environ..

